# Tranylcypromine, a lysine-specific demethylase 1 (LSD1) inhibitor, suppresses lesion growth and improves generalized hyperalgesia in mouse with induced endometriosis

**DOI:** 10.1186/s12958-016-0154-0

**Published:** 2016-04-09

**Authors:** Qunyan Sun, Ding Ding, Xishi Liu, Sun-Wei Guo

**Affiliations:** Cixi Child and Maternal Hospital, 1288 Er’Zhaotan Road, Baishalu, Cixi, Zhejiang China; Shanghai Obstetrics and Gynecology Hospital, Fudan University, 419 Fangxie Road, Shanghai, 200011 China; Shanghai Key Laboratory of Female Reproductive Endocrine-Related Diseases, Fudan University, Shanghai, China

**Keywords:** Endometriosis, Epithelial-mesenchymal transition, Generalized hyperalgesia, Hotplate latency, Lysine-specific demethylase 1, Mouse, Tranylcypromine

## Abstract

**Background:**

Growing evidence indicates that endometriosis is an epigenetic disease. Encouragingly, histone deacetylases (HDACs) and DNA methyltransferases have been shown to be promising targets by numerous in vitro studies. However, only a few studies have shown promising effects of HDAC inhibition in preclinical studies in endometriosis. While lysine-specific demethylase 1 (LSD1) is recently found to be aberrantly expressed in endometriosis, and that the treatment of endometriotic stromal cells with tranylcypromine (TC), an LSD1 inhibitor, significantly reduced cellular proliferation, cell cycle progression, and invasiveness, the in vivo effect of TC treatment is currently lacking. This study sought to evaluate the effect of TC in a mouse model of endometriosis.

**Methods:**

Forty-seven female C57BL/6 mice were used in this experimentation. All mice, except those randomly selected to form Sham surgery (M) and specificity control (S) groups, received an endometriosis-inducing surgery. Group S was set up mainly to ensure that the reduced generalized hyperalgesia in mice treated with TC is not due to any possible analgesic effect of TC, but rather resulting from the treatment effect specific to endometriosis. Two weeks after the surgery, mice that received surgery were further divided randomly into 3 groups: 1) untreated group (U); 2) low-dose TC group (L); 3) high-dose TC group (H). Group S received the same treatment as in group H. Two weeks after treatment, all mice were sacrificed and their ectopic endometrial tissues were harvested and analyzed by immunohistochemistry analysis. Hotplate test was administrated to all mice before the induction, treatment and sacrifice. Lesion size, hotplate latency, immunoreactivity against markers of proliferation, angiogenesis, H3K4 methylation, and of epithelial-mesenchymal transition (EMT).

**Results:**

TC treatment significantly and substantially reduced the lesion size and improved generalized hyperalgesia in a dose-dependent fashion in mice with induced endometriosis. In addition, TC treatment resulted in reduced immunoreactivity to biomarkers of proliferation, angiogenesis, and H3K4 methylation, leading to arrested EMT and lesion growth.

**Conclusion:**

In light of our previously reported reduced cellular proliferation, cell cycle progression and invasiveness resulting from the LSD1 inhibition in in vitro studies, our data strongly suggest that LSD1 is a promising therapeutic target for endometriosis.

**Trial registration:**

Not applicable.

## Background

Endometriosis, characterized by the deposition and growth of functional endometrial-like tissues outside the uterine cavity, is an estrogen-dependent disease, affecting roughly 6–10 % of women of reproductive age [1]. It is a leading cause of disability in women of reproductive age and a major contributing cause for dysmenorrhea, pelvic pain and subfertility [2], impacting negatively on their quality of life [3]. Despite extensive research, the development of effective and safe drugs for endometriosis treatment has been frustratingly slow [4].

Endometriosis is, first and foremost, a hormonal disease, characterized by the increased local production of estrogens due to molecular aberrations in steroidogenesis [5]. It is also recognized as a inflammatory condition [6], featuring overexpression of inflammatory genes, the release of pro-inflammatory cytokines [7, 8], the activation of NF-κB [9–11], and the infiltration of macrophages and lymphocytes [12–14].

Since the report, published over a decade ago, of HOXA10 hypermethylation in eutopic endometrium from women with endometriosis, which ventured the view that “endometriosis may also be an epigenetic disease” [15], accumulating evidence provides strong support for this view [16–19]. Encouragingly, drugs (the so-called “epi-drugs”) targeting aberrant but reversible epigenetic modifications, mainly DNA methylation and histone deacetylation, have been shown to be of therapeutic potential for treating endometriosis. Specifically, histone deacetylases (HDACs) and DNA methyltransferases (DNMTs) have been shown to be promising targets by numerous in vitro studies [20–24]. In contrast, only a few studies have shown promising effects of HDAC inhibition in preclinical studies in endometriosis [25–27].

Epigenetic regulation of gene transcription is mediated by a diverse family of protein complexes, including chromatin remodeling and transcription factors [28]. The epigenetic landscape is complex, encompassing DNA methylation, histone code, non-coding RNA, nucleosome positioning, and DNA sequence. In particular, histone lysine methylation has recently been emerged as one key determinant of transcriptional regulation [29]. The first protein showing histone lysine (K) demethylase (KDM) activity identified in mammals is lysine-specific demethylase 1 (LSD1 or KDM1A) [30]. LSD1 is a flavin-containing amino oxidase (AO) that specifically demethylates mono- or di-methylated lysine 4 at histone H3 (H3K4me1 and H3K4me2) via a flavin adenine dinucleotide (FAD)-monoamine oxidase (MAO) mechanism [31], and, as such, can be inhibited by compounds related to the MAO inhibitor (MAOI) class of pharmaceuticals [32]. LSD1 is intimately involved in DNA methylation [33], interact with nuclear hormone receptors such as estrogen receptor [34], have a potential role in the repression of E-cadherin and thus in the epithelial-mesenchymal transition (EMT) [35] and in the regulation of VEGF [36]. It also interacts closely with HDACs to suppress gene expression [30]. In endometriosis, several lines of evidence implicate the involvement of LSD1. First, a global H3K4 and H3K9 hypomethylation in endometriotic lesions has been reported recently [37]. In addition, global H3 hypoacetylation as well as HDAC1 overexpression also have been reported [17, 38, 39]. While selective inhibition of prostaglandin E2 receptors EP2 and EP4 is shown to have many desirable therapeutic effects [40], it has no effect on LSD1 expression in endometriotic lesions [41].

We have recently shown that LSD1 gene and protein expression is elevated in endometriosis, and treatment of endometriotic stromal cells with tranylcypromine (TC), an LSD1 inhibitor, significantly reduced cellular proliferation, cell cycle progression, and invasiveness [42]. However, the in vivo effect of TC treatment is currently lacking. In light of scant, if any, preclinical studies on the use of epi-drugs other than HDAC inhibitors in endometriosis, this study sought to evaluate the effect of TC in a mouse model of endometriosis.

## Methods

### Mouse experiment

Forty-seven virgin female C57BL/6 mice, 8 weeks old and about 18–20 g in weight, were purchased from Shanghai BiKai Laboratory Animal Center (Shanghai, China) and used for this study. They were housed individually in cages, maintained under controlled conditions with a light/dark cycle of 12/12 h, and had access to chows and water *ad libitum*. All experiments in this study were performed under the guidelines of the National Research Council’s *Guide for the Care and Use of Laboratory Animals* and approved by the institutional experimental animals review board of Shanghai OB/GYN Hospital, Fudan University.

After 3 days of acclimatization and before the surgery (see Surgical procedures below), a baseline hotplate test (Test 1) was administrated to all mice as reported previously [25]. Thereafter, all mice, except 7 and 9 that were randomly selected to form groups M (sham surgery) and S (for specificity control), respectively, received an endometriosis-inducing surgery. Two weeks after the surgery, the 31 mice that received surgery were further divided at random into 3 groups: 1) untreated (U) group (*n* = 11); 2) low-dose TC (L) group (*n* = 10); 3) high-dose TC (H) group (*n* = 10). Mice in group M received similar care as others but received a sham surgery (fragments of fat tissues, instead of endometrial tissue fragments). The set-up of group M was meant to validate the results obtained from humans, and their endometrial tissue samples were subjected to immunohistochemistry analysis of LSD1, using the same LSD1 primary antibody used in humans. They were sacrificed 2 weeks after surgery.

**Fig. 1:**
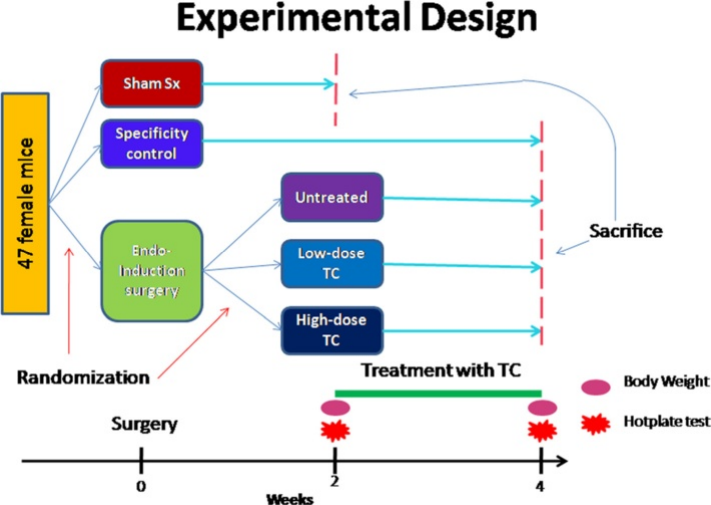
Schematic illustration of the experimental design of this study. Some abbreviations used in the figure: Endo-induction surgery, endometriosis-induction surgery; Sham, sham surgery, a surgery that transplant fragments of fat tissues, instead of endometrial tissue fragments, to the peritoneum of the pelvic cavity and the mesenterium of the small intestine; Sx, Surgery; TC, tranylcypromine

Two weeks after induction, mice in group U received 0.3 mL intra-peritoneal (i.p.) injection of sodium sulfate solution (a solvent for TC) every two days, while mice in groups L and H received i.p. injection of 1 or 2 mg/kg bodyweight TC dissolved in 0.3 ml sodium sulfate solution every two days, respectively. Meanwhile, group S received a high-dose TC treatment in identical fashion as to group H. The dose in group H was about the half of the dose used in a cancer study [43]. Group S was set up mainly to ensure that the reduced generalized hyperalgesia, if any, in mice treated with TC is not due to any possible analgesic effect of TC, but rather resulting from the treatment effect specific to endometriosis. All groups received identical care except the administration of different types and/or doses of drugs. The schematic illustration of the experimental design is shown in Fig. 1.

To gauge the change in thermal latency as a result of induced endometriosis, a second round of hotplate test (Test 2) was administrated to all mice two weeks after surgery and before the treatment. Two weeks after treatment, the third round of hotplate test (Test 3) was administrated to all mice. Then the mice were sacrificed through cervical dislocation. The abdominal cavity was immediately reopened through the original incision, and all lesions were measured with two perpendicular diameters (the length *D*_*1*_ and the width *D*_*2*_). The surface area of each endometrial implant tissue was calculated by the formula: *S = π × D*_*1*_ *× D*_*2*_/4 (in mm^2^). The total surface area of ectopic lesions in each mouse was evaluated. Ectopic lesion tissues in groups H, L and U were harvested and fixed immediately after collection in 10 % formalin and embedded in paraffin for histopathologic examination and immunohistochemical analysis.

### Surgical procedures

Anaesthetized with 300 mg/kg chloral hydrate, surgery was performed aseptically to auto-transplant small pieces of uterine to the peritoneum of the pelvic cavity and the mesenterium of the small intestine, as described previously [25]. Laparotomy was performed and the left uterine horn was removed. The excised horns were immersed in a sterile lactate solution and opened longitudinally. Each uterine segment was then cut into four smaller, roughly equal-sized (2.5 mm in diameter) fragments. A total of 4 uterine fragments were sutured to the peritoneal wall of the lower part of the lateral abdominal and pelvic cavity with a 7/0 braided silk suture. Then the midline incision was closed with a 3/0 braided silk suture. After surgery, all mice were administrated with 40,000 U of penicillin i.m. to prevent infection. In addition, they received subcutaneous injection of 0.2 mg/kg bodyweight 17β-estradiol solution twice a week for the next 2 weeks to stimulate and maintain the growth of endometrial implants [25]. Endometriosis was successfully induced in all mice.

### Immunohistochemistry and histochemistry

Mice ectopic lesions were fixed for 24 h at room temperature with 10 % formalin neutral buffer solution. After fixation, the tissues were placed in 70 % ethanol overnight at 4 °C, then embedded in paraffin and sectioned at 4-μm thickness. For each paraffin-embedded tissue block, the first resultant slide was stained for H&E to confirm pathologic diagnosis, and the subsequent slides were stained for proliferating cell nuclear antigen (PCNA) (a marker for cellular proliferation), vascular endothelial growth factor (VEGF), CD31 (for counting microvessel density or MVD), H3K4me1, H3K4me2, E-cadherin and vimentin. Routine deparaffinization and rehydration procedures were performed following published protocols.

For antigen retrieval, the slides were heated at 98 °C in the EDTA buffer (pH 8.0) or the citric acid solution (pH 6.0) (depending on the primary antibody used) for a total of 30 min and cooled naturally at room temperature. The rabbit polyclonal antibodies against PCNA (Thermo Littleton, CO, USA), H3K4me1 (Abcam, Cambridge, MA, USA), H3K4me2 (Abcam), VEGF (Santa Cruz, TX, USA), CD31 (Abcam), E-cadherin (CST, MA, USA), vimentin (Abcam) diluted to 1:100, 1:100, 1:100, 1:50, 1:50, 1:100 and 1:200, respectively, were used as primary antibodies. Normal control mouse endometrial tissues in group M and the ectopic endometrial tissues in group U were also incubated with a rabbit polyclonal antibody against LSD1 (1:100; Novus Biologicals, Littleton, CO,USA) at 4 °C overnight. After slides were rinsed by PBS, they were incubated at room temperature for 15 min and 10 min, respectively, in biotinylated secondary antibody and treptavidin-labeled HRP (MR-SPR120, Shanghai MingRui BioTech, Shanghai, China). Finally, the sections were stained for 2 min or until appropriate for microscopic examination with diaminobenzidine. The sections were then counter-stained with haematoxylin, dehydrated in a graded alcohol series, cleared in xylene, and finally mounted in balsam. Human invasive breast cancer tissue samples were used as positive control. Negative control sections were treated identically, except that the specific antibody was replaced by the same concentration of normal rabbit serum. No positive reaction was observed under these conditions. Since TC blocks LSD1 activity [44], immunohistochemistry analysis of LSD1 in treated mice was not performed.

The number and intensity of positive cells were counted by Image Pro-Plus 6.0 (Media Cybernetics, Inc., Bethesda, Maryland, USA). Images were obtained with the microscope (Olympus BX51, Olympus, Tokyo, Japan) fitted with a digital camera (Olympus DP70, Olympus, Tokyo, Japan). A series of 3–5 random images on several sections were taken for each immunostained parameter to obtain a mean value. Staining was defined via color intensity, and a color mask was made. The mask was then applied equally to all images, and measurement readings were obtained. Immunohistochemical parameters assessed in the area detected included (a) integrated optical density (IOD); (b) total stained area (S); and (c) mean optical density (MOD), which is defined as MOD = IOD/S, equivalent to the intensity of stain in all positive cells.

### Statistical analysis

The comparison of distributions of continuous variables between or among two or more groups was made using the Wilcoxon’s and Kruskal’s test, respectively, and the paired Wilcoxon test was used when the before-after comparison was made for the same group of subjects. Pearson’s or Spearman’s rank correlation coefficient was used when evaluating correlations between two variables when both variables were continuous or when at least one variable was ordinal. To see whether TC treatment and other possible factors were responsible for the change in hotplate latency before and after the treatment, a multiple linear regression model was used. The treatment of 0, 1 mg/kg, and 2 mg/kg TC was coded as 0, 1, and 2, respectively.

To further characterize the treatment effect, we carried out a hierarchical cluster analysis with scaled data and the Euclidean distance as the similarity metric, with the average linkage being the clustering method. The resulting dendrogram was represented as a heatmap. A multidimensional scaling (MDS) analysis was performed to discriminate all mice used in this study.

*P* values of less than 0.05 were considered statistically significant. All computations were made with R 3.2.2 [45] (www.r-project.org).

## Results

Before the treatment was started, two mice in group L died at the 7th day after surgery following the 17β-estradiol injection, and 2 mice each in groups S and H were found dead at the 5th day after the treatment was started. An autopsy ensued for all dead mice, but no apparent abscess, hemorrhage, obstruction, or embolus was found. The respiratory, circulation, and urinary systems, and the liver and spleen all appeared normal. Since mice in groups H and S all received higher TC dosage, it is likely that the toxicity might have resulted in the demise. Consequently, groups M, U, L, H and S had 7, 11, 8, 8 and 7 mice, respectively at the end of the experiment and were used in the following analyses. In the L group, TC appeared to be well tolerated, and no adverse event was observed.

### Inhibition of LSD1 results in reduced lesion size and improved hyperalgesia in mice with induced endometriosis

**Fig. 2:**
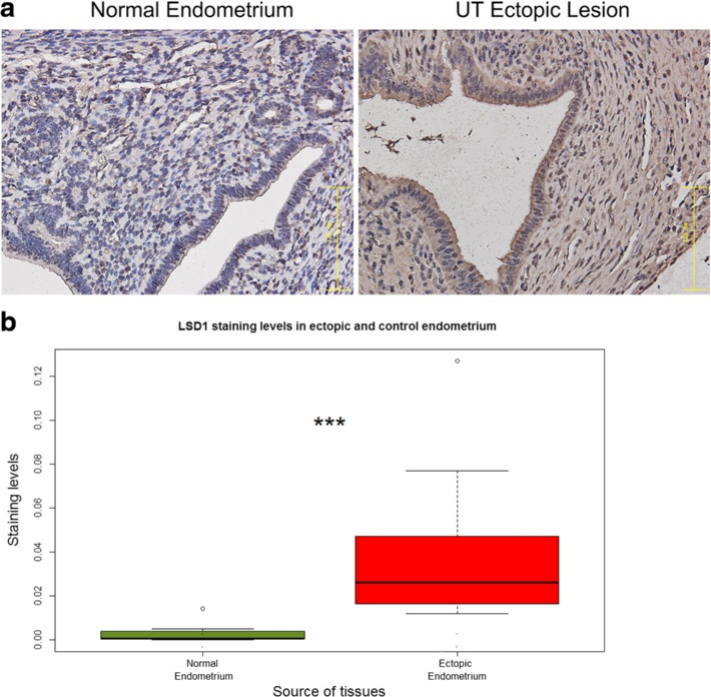
**a** Representative photo micrographs showing immunoreactivity to LSD1 in the endometrium from a mouse that received a sham surgery and from a mouse that had induced endometriosis but received no treatment. Magnification: X400. The scale bar represents 125 μm. **b** Boxplot of LSD1 staining levels in normal endometrium from mice in group SHAM and in ectopic endometrium from mice in group U. “***” denotes the *p*-value of the difference between the two groups is less than 0.001

We first show that, compared with the control endometrium from mice that received sham surgery (and thus had no endometriosis), the LSD1 staining levels in endometriotic lesions in group U mice were significantly elevated (*p* = 4.4 × 10^−4^; Fig. 2), similar to what we found in humans [42].

**Fig. 3:**
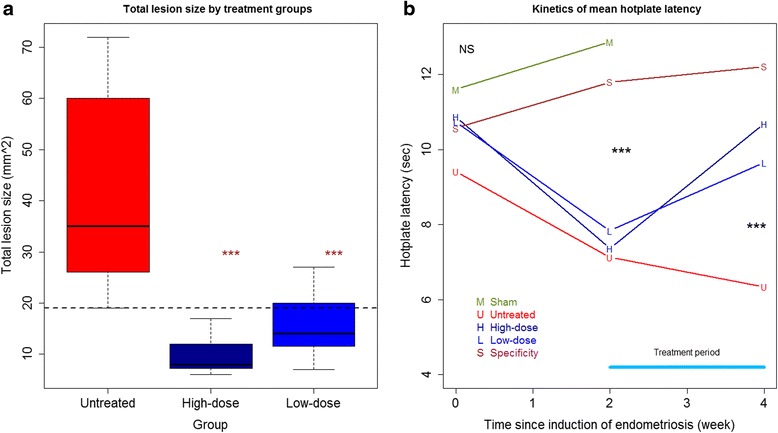
**a** Boxplot of lesion size in different treatment groups. “***” denotes the p-values of the difference between the designated group and the untreated group are less than 0.001. **b** The time-course of the mean hotplate latency in different treatment groups. Abbreviations used in the figure: M, sham surgery group; U, untreated mice; L, mice treated with low-dose TC; H, mice treated with high-dose TC; S, specificity control group

We also found that TC treatment dose-dependently reduced the lesion size (Fig. 3a; *p* = 1.8 × 10^−7^, *R*^*2*^ = 0.67, by a multiple linear regression analysis with log-transformed lesion size). In fact, low- and high-dose TC treatment resulted in an average of 62.4 and 76.7 % reduction in lesion size, respectively (*p* = 0.0013 and *p* = 0.0003, respectively; Fig. 3a).

As expected, there was no difference in hotplate latency prior to the induction of endometriosis (*p* = 0.16; Fig. 2b). However, 2 weeks after the induction but before the TC treatment, a significant difference in hotplate latency among the 5 groups of mice was found (*p* = 0.0001; Fig. 3b). In fact, mice with induced endometriosis had a significantly decreased latency as compared with those without (*p* = 2.1 × 10^−6^; Fig. 3b), consistent with what we reported previously [25–27]. Two weeks after treatment, there was a significant difference in hotplate latency among the 4 groups of mice (*p* = 0.0003; Fig. 3b; Hotplate latency in the sham group was not measured). In fact, mice with endometriosis that received TC treatment had significantly longer latency—in a dose-dependent manner---than those without (*p* = 0.0003, by multiple linear regression controlling for before-treatment levels and the bodyweight at sacrifice, *R*^*2*^ = 0.87; Fig. 3b). This can be seen from the improved latency in mice with endometriosis and treated with TC but not in untreated mice (Fig. 3b). The mice in group S, on the hand, had no significant change in latency (*p* = 0.40; Fig. 3b), suggesting that the improvement in hotplate latency in TC-treated mice was endometriosis-specific.

### Inhibition of LSD1 results in reduced proliferation and angiogenesis in ectopic endometrium

**Fig. 4:**
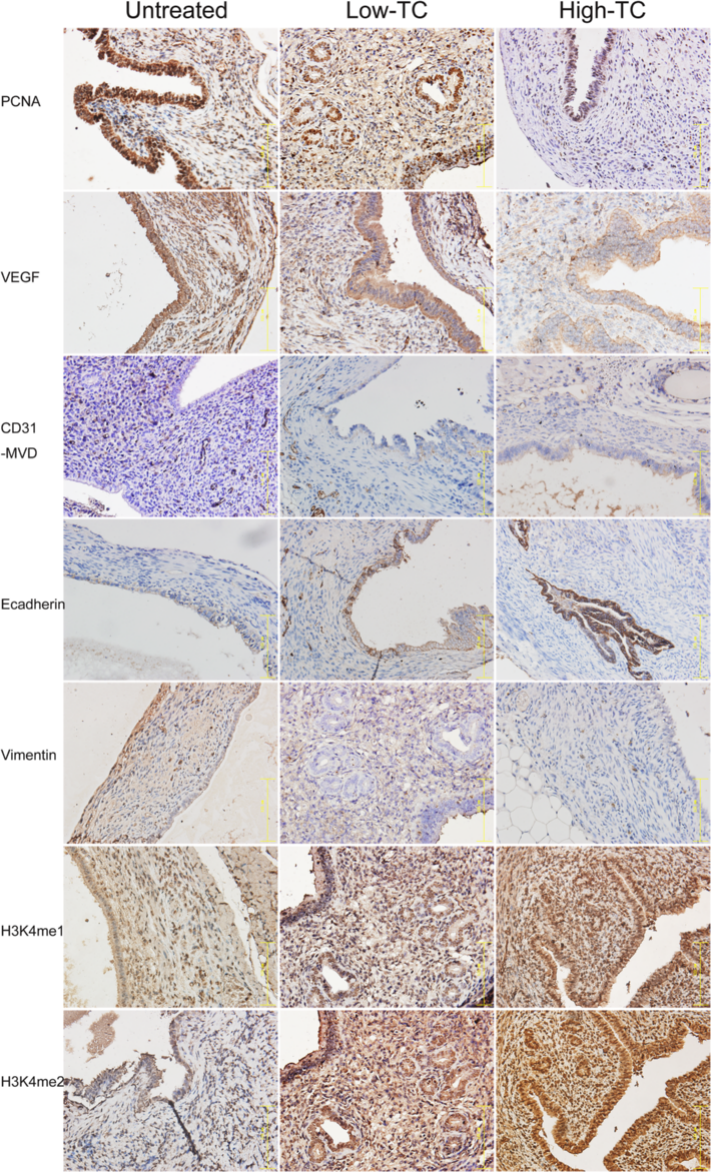
Representative micrographs showing immunoreactivity to PCNA, VEGF, CD31 (MVD), E-cadherin, vimentin, H3K4me1, and H3K4me2, in ectopic endometrium among different groups. The abbreviations used: Low-TC, tissue sample taken from a mouse treated with low-dose TC; High-TC, treated with high-dose TC; UT, untreated. Magnification in all figures: X400. The scale bar represents 125 μm

We further performed an immunohistochemistry analysis of PNCA, VEGF, CD31, H3K4me1, H3K4me2, E-cadherin, and vimentin for ectopic endometrium. We found that PCNA, H3K4me1 and H3K4me2 staining was seen in cellular nuclei in both the stromal and epithelial cells of the ectopic endometrium, while VEGF immunoreactivity was seen mostly in the cytoplasm of glandular epithelial cells as well as of vascular endothelial cells (Fig. 4). As expected, CD31 staining was seen mostly in vascular endothelial cells, and E-cadherin staining was seen mostly in cell membranes of glandular epithelium. On the other hand, vimentin staining was seen in the cytoplasm of stromal cells (Fig. 4).

**Fig. 5:**
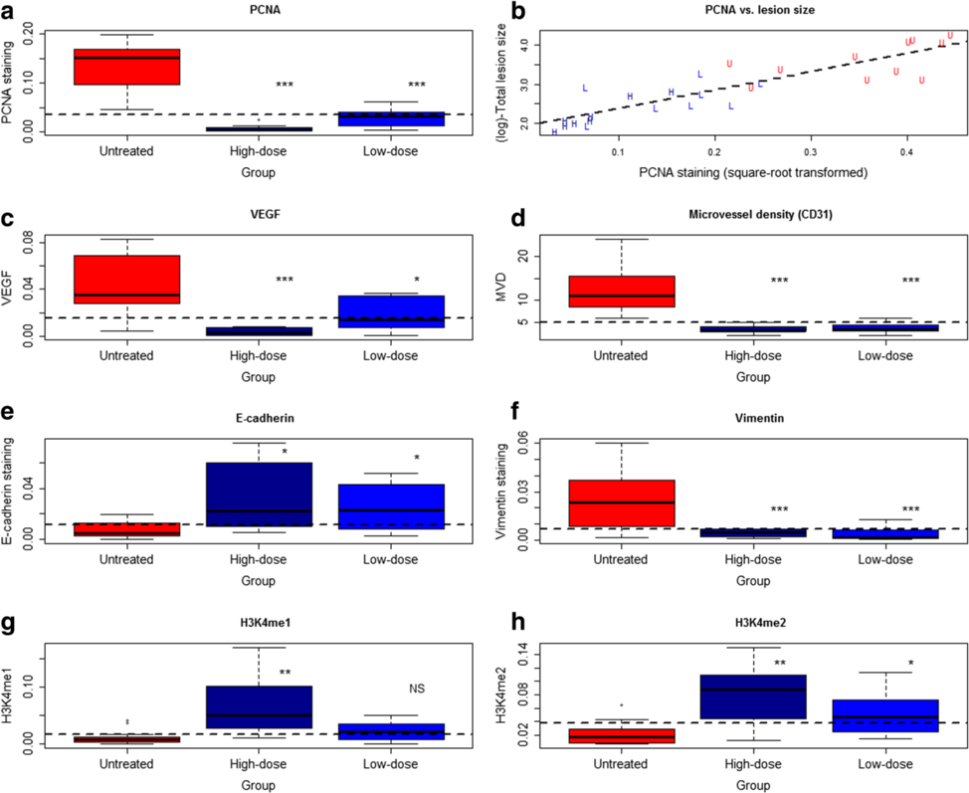
**a** Boxplot of PCNA staining levels in different groups of mice. **b** Scatter plot of lesion size vs. the PCNA staining levels in ectopic endometrium. The dashed line represents the regression line. Each alphabet represents one data point, where U, L, and H denote that the mouse was from group UT, L, and H, respectively. The numbers in the figures are p-values for statistical significance in testing the difference among different groups (Kruskal-Wallis test). **c** Boxplot of VEGF staining levels in different groups of mice. **d** Boxplot of the number of CD31-positive microvessels in different groups of mice. **e** Boxplot of E-cadherin staining levels in different groups of mice. **f** Boxplot of vimentin staining levels in different groups of mice. **g** Boxplot of H3K4me1 staining levels in different groups of mice. **h** Boxplot of H3K4me2 staining levels in different groups of mice. ‘*’, *p* < 0.05; ‘**’, *p* < 0.01; ‘***’, *p* < 0.001. denoting the statistical significance levels as compared with the untreated group

We found that the TC dosage correlated closely with the immunoreactivity to all these proteins. In particular, the TC dosage correlated negatively with staining of PCNA (*r* = −0.87, *p* = 2.7 × 10^−9^; Spearman’s correlation), VEGF (*r* = −0.75, *p* = 7.9 × 10^−6^), MVD (CD31) (*r* = −0.80, *p* = 4.3 × 10^−7^), and vimentin (*r* = −0.52, *p* = 0.0054), but positively with that of E-cadherin (*r* = 0.55, *p* = 0.0029), H3K4me1 (*r* = 0.64, *p* = 0.0004), and H3K4me2 (*r* = 0.63, *p* = 0.0005). For all markers, there was a significant difference in immunoreactivity among the 3 groups (all *p*-values < 0.015; Fig. 5a, c-h). Precisely as expected, TC treatment resulted in elevated H3K4me1 and H3K4me2 staining levels (Fig. 5g, h). Linear regression analyses indicated that TC dosage was negatively associated with the decrease in immunoreactivity to PCNA (*p* = 2.8 × 10^−7^, *R*^*2*^ = 0.76; square-root transformed to improve normality; Fig. 5a), VEGF (*p* = 5.2 × 10^−6^, *R*^*2*^ = 0.57; Fig. 5c), MVD (*p* = 1.5 × 10^−6^, *R*^*2*^ = 0.61; Fig. 5d), and vimentin (*p* = 0.0026, *R*^*2*^ = 0.31; Fig. 5f) but positively associated the increase in immunoreactivity to E-cadherin (*p* = 0.0024, *R*^*2*^ = 0.31; Fig. 5e), H3K4me1 (*p* = 0.0005, *R*^*2*^ = 0.39; Fig. 3g), and H3K4me2 (*p* = 0.0004, *R*^*2*^ = 0.39; Fig. 5h).

As expected, the lesion size correlated positively with the PCNA staining levels (*r* = 0.87, *p* = 3.5 × 10^−9^, both square-root transformed; Fig. 5b). The PCNA immunoreactivity correlated positively with the MVD (*r* = 0.64, *p* = 0.0003), which, in turn, positively correlated with that of VEGF (*r* = 0.67, *p* = 0.0001). The VEGF staining levels correlated negatively with that of both H3K4me1 (*r* = −0.42, *p* = 0.029) and H3K4me2 (*r* = −0.49, *p* = 0.0089).

### Hierarchical cluster analysis of measured phenotypes and IHC immunoreactivity

**Fig. 6:**
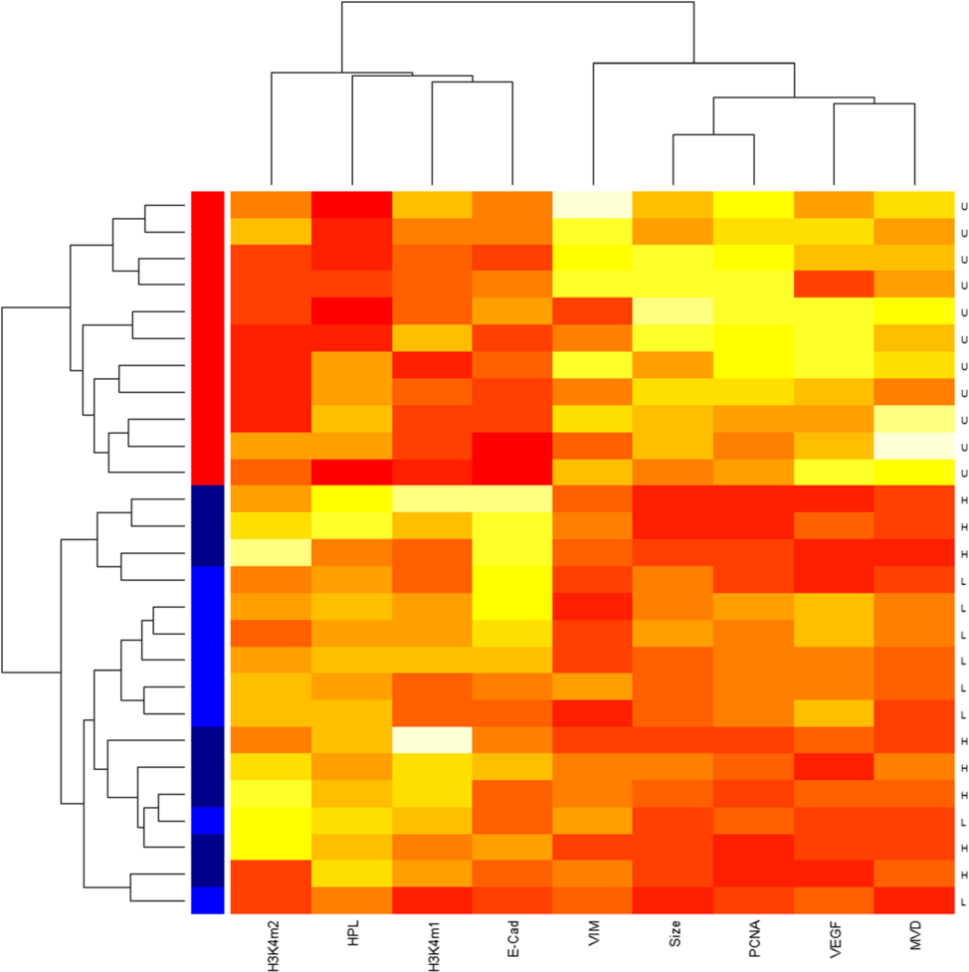
Hierarchical clustering heatmap of phenotypes/immunoreactivity measurements and mice. The heatmap was organized by clustering both mice (by rows) and measured phenotypes/immunoreacivity measurements (by columns). The red color represents the minimal values while the white color represents the maximal values. The alphabets on the rightmost panel represents which treatment group the mouse came from, where the group abbreviations are the same as in Fig. 5. The labels in the bottom are the names of the phenotypes/immunoreactivity measurements. HPL: hotplate latency. The color strip on the left: The violet color indicates mouse from the untreated group, the blue color, the low-dose TC group, and the dark blue color, the high-dose TC group

To gain more insights into the possible mechanisms of TC treatment effect, we performed a hierarchical cluster analysis of all measured phenotypes and IHC immunoreactivity measurements, all square-root transformed, and the results are presented as a heatmap (Fig. 6). We can see from Fig. 6 that all mice could be grouped roughly into 2 clusters: cluster 1 is featured by a high growth propensity, namely, larger lesion size, and elevated immunoreactivity to VEGF, PCNA, vimentin and higher MVD in the ectopic endometrium. In addition, it is featured by shorter hotplate latency and reduced immunoreactivity to H3K4me1/me2 and E-cadherin. This cluster includes exclusively all mice in the untreated group. Cluster 2 is the opposite, and is characterized by a reduced growth propensity and longer latency, heavier bodyweight and elevated H3K4me1/me2 and E-cadherin staining. This cluster includes exclusively all mice that received TC treatment, although mice in the low- and high-dosage groups appeared to overlap (Fig. 6).

**Fig. 7:**
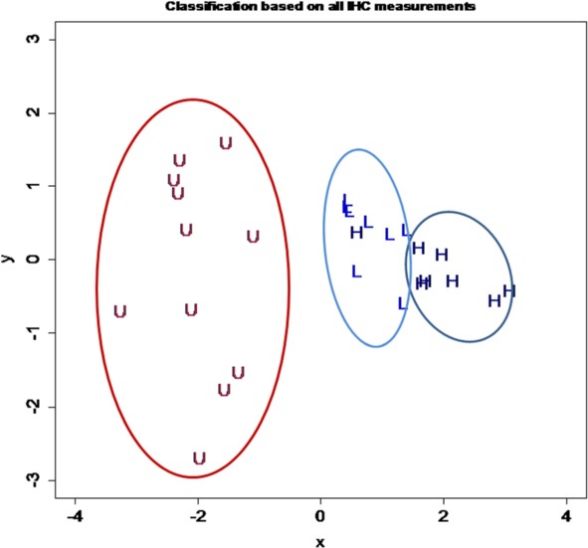
Results of multidimensional scaling analysis using all immunoreactivity data. All mice were represented by their group labels, which are the same as used in Fig. 2b

From the heatmap, it seems that those proteins involved in growth propensity can be clustered together, which forms a greater cluster with the lesion size (Fig. 6). In contrast, hotplate latency, E-cadherin and H3K4me1/me2 staining levels formed another group (Fig. 6). Consequently, we used these staining levels and MVD scores to carry out a multidimensional scaling analysis. The result is shown in Fig. 7. We can see that, indeed, the use of these measurements can cluster the mice very nicely, almost a perfect match with the original treatment assignment (Fig. 7).

## Discussion

In this study, we have demonstrated that TC treatment significantly and substantially reduced the lesion size and improved generalized hyperalgesia in a dose-dependent fashion in mice with induced endometriosis. In addition, TC treatment results in reduced immunoreactivity to VEGF, PCNA, and vimentin but elevated E-cadherin and H3K4me1/me2 staining levels, leading to arrested EMT and decreased MVD as well as lesion growth. This, in conjunction with our previously reported reduced cellular proliferation, cell cycle progression and invasiveness resulting from the LSD1 inhibition in vitro studies [42], strongly suggests that LSD1 is a promising therapeutic target for endometriosis.

Endometriotic epithelial cells are known to have increased invasive propensity [46, 47], which is characterized with decreased E-cadherin expression [47], likely due to its promote hypermethylation [48]. The loss of E-cadherin expression is a hallmark of EMT, a highly conserved cellular process that allows polarized and generally immotile epithelial cells to convert to motile mesenchymal cells, and occurs in embryonic development, cancer, and as a physiological response to injury [49]. EMT endows cells with migratory and invasive capabilities, increasing the invasive propensity.

EMT is now well-documented in endometriosis [50–53], featuring decreased E-cadherin expression but increased vimentin expression in the epithelial component of endometriotic lesions. Several transcription factors known to play critical roles in EMT, such as Snail, Twist and ZEB1 [49], are also reported to be upregulated in endometriosis [52]. Yet Snail uses its SNAG domain as a molecular hook to associate the LSD1/HDAC complex [54]. Once recruited to the E-cadherin promoter, the complex may modify histones to generate a repressive chromatin environment featuring H3K4 hypomethylation and H3/H4 hypoacetylation [55]. Our data appear to be consistent with published data of H3, but not H4, hypoacetylation [38] and global H3K4 hypomethylation [37] and the increased LSD1 expression [42] in endometriosis.

Our data are also consistent with the abrogated EMT as a result of LSD1 inhibition through TC treatment, as seen by reduced vimentin staining but increased E-cadherin staining in the epithelial component of lesions as well as increased H3K4me1 and H3K4me2 staining levels in endometriotic lesions. The increased expression of E-cadherin, a marker of epithelial cells, and the decreased expression of vimentin, a marker of mesenchymal cells, seen in mice treated with TC is consistent with reports that LSD1 is essential for Snai1-mediated transcriptional repression and for maintenance of the silenced state of Snai1 target genes, including E-cadherin, in invasive cancer cells [35], and that chromatin reprogramming during EMT is LSD1-dependent [56]. Inhibition by TC also is found to suppress Slug-LSD1 interaction, blocking cancer cell motility and invasion [57]. Since loss of E-cadherin is intimately related with EMT and invasiveness, even in endometriosis [48, 51], these results appear to give more credence to the reduced invasiveness of endometriotic stromal cells treated with TC (Fig. 5).

The elevated LSD1 staining levels in ectopic implants as compared with normal endometrium are also consistent with our human data [42]. The reduced VEGF expression is consistent with the report that functional deletion of LSD1 impairs VEGF transcription [36], and also is consistent with the reduced MVD, and subsequently PCNA staining levels and lesion size.

Our data demonstrate a potent therapeutic effect of LSD1 inhibition by TC, as evidenced by close to 2/3 reduction in lesion size in low-dose group and over 3/4 of reduction in high-dose group. Aside from the abrogation of the EMT process and thus invasiveness in the development of endometriosis, the decreased angiogenesis and cellular proliferation, LSD1 suppression by TC may also have other desirable therapeutic effects, yet to be identified, since the H3K4 and H3K9 demethylation is likely to be global. For example, as a nonselective and irreversible MAO inhibitor, TC is anti-depressant and anxiolytic (used only in Germany). After all, women with endometriosis, especially those with dysmenorrhea and infertility, may experience anxiety [58]. It is also possible that its use, in combination with other therapeutics such as HDAC inhibitors, may generate synergistic therapeutic effect as in acute myeloid leukemia [59].

Given the cross-talks between LSD1 and HDACs [30, 60], it is not surprising to see that the use of LSD1 inhibitors may sensitize cancer cells to HDAC inhibitors [61]. HDAC inhibition also has been shown to stimulate H3K4 methylation through suppression of KDMs including LSD1 [62]. This, in conjunction with the data presented here and elsewhere, suggests that LSD1 is promising candidate for targeted therapy to treat endometriosis.

However, caution also should be exercised. Endometriosis is largely a benign disease and certainly is not life-threatening. As such, it places higher premium on drug safety as compared with other life-threatening diseases such as cancer. While LSD1 suppression by TC may hold promises in treating endometriosis, the high mortality in mice that received high-dose of TC raises a red flag. It is also conceivable that H3K4 demethylation may be part of the normal physiology, and, as such, TC treatment may have off-target toxicity. Whether TC should be used in treating human endometriosis, dosage, formulation, and route of delivery surely will need more investigation and a thorough evaluation of the benefit versus risk ratio. The encouraging results shown in this study merely demonstrate the possibility of targeting LSD1 for therapeutic purpose. They are not meant to advocate TC per se for the treatment of human endometriosis. Encouragingly, several LSD1 inhibitors are on the horizon [63].

## Conclusions

In summary, we found that tranylcypromine treatment results in marked changes that are consistent with retarded EMT, decreased angiogenesis and lesion growth, and improved hyperalgesia in mice with induced endometriosis, along with increased H3K4 methylation. Taken together, these results suggest that LSD1 may be a promising therapeutic target for endometriosis.

## Abbreviations

AO: amino oxidase
DNMT: methyltransferase
EMT: epithelial-mesenchymal transition
FAD: flavin adenine dinucleotide
H3K4me1: mono-methylated lysine 4 at histone H3
H3K4me2: di-methylated lysine 4 at histone H3
HDAC: histone deacetylase
LSD1: lysine-specific demethylase 1
MAO: monoamine oxidase
MDS: multidimensional scaling
MVD: microvessel density
PCNA: proliferating cell nuclear antigen
TC: tranylcypromine
VEGF: vascular endothelial growth factor
